# Inhibitory Effects of *Euphorbia ebracteolata* Hayata Extract ECB on Melanoma-Induced Hyperplasia of Blood Vessels in Zebrafish Embryos

**DOI:** 10.1155/2021/5543259

**Published:** 2021-04-26

**Authors:** Wenjing Dong, Xinyue Han, Chao Bao, Saijilahu Tai, Yuxia Bai, Liang Xu, Jingfeng Yang, TinChung Leung, Wuliji Ao, Wu Dong

**Affiliations:** ^1^Inner Mongolia Key Laboratory of Toxicant Monitoring and Toxicant and Toxicology, College of Animal Science and Technology, Inner Mongolia University for Nationalities, Tongliao, Inner Mongolia 028000, China; ^2^Mongolian State University of Education, Ulaanbaatar 210648, Mongolia; ^3^Inner Mongolia Research Institute of Traditional Mongolian Medicine Engineering Technology/College of Mongolian Medicine and Pharmacy, Inner Mongolia University for Nationalities, Tongliao 028000, China; ^4^Inner Mongolia Key Laboratory for the Natural Products Chemistry and Functional Molecular Synthesis, College of Chemistry and Chemical Engineering, Inner Mongolia University for Nationalities, Tongliao, Inner Mongolia 028000, China; ^5^Julius L. Chambers Biomedical Biotechnology Research Institute, Dept of Biological & Biomedical Sciences, North Carolina Central University, Kannapolis, NC 28081, USA

## Abstract

Melanoma is a serious malignant form of skin cancer. Euphorbiaceae compound B (ECB, 2,4-dihydroxy-6-methoxy-3-methylacetophenone) is an acetophenone compound that is isolated from *Euphorbia ebracteolata* Hayata (EEH), an herbaceous perennial, and has antitumor activity. Here, we transplanted human melanoma cells into zebrafish embryos to establish a zebrafish/melanoma model. We showed that this model can be used to evaluate the therapeutic effect of EEH and ECB and discussed its potential mechanism of action. The results showed that ECB was an active ingredient of EEH in inhibiting melanoma-induced hyperplasia of blood vessels in zebrafish embryos, similar to the angiogenic inhibitor vatalanib. ECB inhibited the number and length of subintestinal veins (*p* < 0.05), as well as the distribution of melanoma in zebrafish embryos (*p* < 0.05). More importantly, unlike vatalanib, ECB only inhibited melanoma-induced abnormal and excessive growth of blood vessels in xenografts. In addition, ECB inhibited the mRNA expression of *vegfr2* and *vegfr3* in zebrafish. Both *vegfr2* and *vegfr3* are essential genes that regulate blood vessel formation and upregulate the expression of *p53* and *casp3a* genes in zebrafish. Together, the above-mentioned results indicate that ECB has a potential antimelanoma effect *in vivo*, which may be mediated by inhibiting vascular endothelial growth factor receptors.

## 1. Introduction

Malignant melanoma is the most serious type of skin cancer, which is caused by hyperplasia of melanocytes in the skin. In the body, melanoma cells can rapidly metastasize, and patients with metastatic melanoma have a survival of no more than five years [1]. Euphorbiaceae compound B (ECB, 2, 4-dihydroxy-6-methylbenzoidone) is a compound of benzene ketones in the *Euphorbia ebracteolata* Hayata (EEH) and *Euphorbia fischeriana* Steud [2, 3]. Studies that focused on the anticancer effects of diterpenoids in Euphorbiaceae have been reported; however, studies on ECB are limited. Blocking the pathway underlying tumor angiogenesis may be a unique way to block the growth of blood vessels that support tumor growth [4]. The zebrafish model can be used as a viable model for whole-animal screening using small molecules that affect tumor angiogenesis [5]. In particular, the subintestinal vein (SIV) of zebrafish/tumor xenografts can be visualized noninvasively in real time using vascular-specific transgenic zebrafish or can be stained using blood vessel dye and visualized under a microscope [6]. Koenig et al. showed that *vegfaa* was expressed at the site of intestinal vascularization and might provide a guiding signal and that *vegfc* overexpression could lead to SIV overgrowth. The authors suggested that (1) Vegfaa signaling might regulate endothelial cells to migrate out of the existing vasculature and subsequently merge to form intestinal blood vessels and that (2) Vegfc could induce growth of the SIV [7]. PTK787, a potent angiogenic inhibitor, inhibits vascular endothelial growth factor receptors (VEGFR) and platelet-derived growth factor receptor (PDGFR) tyrosine kinase and blocks cell proliferation and cell survival, resulting in cell death and reduced blood vessel density in leiomyosarcoma cells, as well as in zebrafish xenografts [8, 9]. The potent B-raf inhibitor PLX4720 (PLX) that acts against melanoma reduced the binding of C-MYC to the *VEGF* promoter to reduce the expression of VEGF in melanoma cells and increased the infiltration of adoptively transferred T cells, thereby leading to an antitumor effect [10].

As a model for high-throughput screening of anticancer drugs, zebrafish have been shown to be a potential model. In this study, we used *Tg(flk1:GFP)* transgenic zebrafish as a model to quantify fluorescent blood vessels after transplantation of melanoma cells into zebrafish embryos. We aimed to use this model for screening the antimelanoma effect of ECB through tumor-induced angiogenesis and metastasis. The potential underlying mechanisms involved were also studied (see Graphic abstract).

## 2. Materials and Methods

### 2.1. Chemicals and Reagents

Human melanoma cells (A2058 cells), originated from a 43-year-old Caucasian adult male, were obtained from the American Type Culture Collection (ATCC, Manassas, VA, USA). Human umbilical vein endothelial cells (HUVECs) were extracted from human umbilical cord vein tissue and obtained from ScienCell (San Diego, CA, USA). The Vybrant™ CM-DiI cell-labeling solution (CM-DiI) was purchased from Invitrogen (Carlsbad, CA, USA). Vatalanib (PTK) and PLX-4720 (PLX) were purchased from MedChemExpress (Monmouth Junction, NJ, USA). ECB was purchased from the National Institutes for Food and Drug Control (Beijing, China). Other compounds and reagents were purchased from Sigma-Aldrich (St. Louis, MO, USA).

### 2.2. Preparation and High-Performance Liquid Chromatography Analysis of EEH

*Euphorbia ebracteolata* Hayata (EEH) was provided by Liang Xu's Lab, and EEH was authenticated and analyzed by Dr. Liang Xu from the Inner Mongolia Key Laboratory for the Natural Products Chemistry and Functional Molecular Synthesis, College of Chemistry and Chemical Engineering, Inner Mongolia University for Nationalities (Tongliao, China). A total of 10 g EEH powder was soaked into 100 mL of 90% ethanol at room temperature for 24 hours. The supernatant was concentrated to 10 mL after the mixture was refluxed for 3 hours, filtered through a 0.22 *µ*m filter, and used as a 1 g/mL stock solution. ECB is a major ingredient of EEH (Figure S1) [11].

### 2.3. Zebrafish Breeding

*Tg(flk1:GFP)* zebrafish were donated by the Chinese Academy of Sciences and raised in a zebrafish breeding system (Environ Science, Beijing, China) at a water temperature of 28.5°C and a 14 : 10-hour day-night cycle. The zebrafish breeding and testing process was approved by the Animal Protection Association of Inner Mongolia University for Nationalities (Tong Liao, China).

### 2.4. Establishment of a Zebrafish/Melanoma Xenograft Model

Melanoma cells were labeled with a fluorescence dye (CM-DiI), and zebrafish/melanoma xenografts were cultured in a 35°C incubator for 2 h, then transferred to a 28.5°C incubator until 24 hours after injection (hpi) and 48 hpi. After 6 h of culture (6 hpi), zebrafish/melanoma xenografts that showed the same amount of melanoma cells under a fluorescence microscope were selected for further analysis.

### 2.5. EEH or ECB Treatment and Calculation of Blood Vessel Number, Length, and CM-DiI Labeling (Melanoma Cells) Area in Zebrafish

Zebrafish embryos were imaged using an inverted fluorescence microscope (Olympus IX73, Tokyo, Japan) at 24 hpi to capture SIV blood vessels and melanoma fluorescence in zebrafish embryos. ImageJ software (NIH, Bethesda, MD, USA) was used to measure the length of SIV ectopic blood vessels, the CM-DiI labeling area, and the number of ectopic blood vessels. After imaging at 24 hpi, 10 zebrafish xenografts were placed in a 6-well plate and treated with EEH, ECB, PTK, PLX, or buffer for the control group. Zebrafish xenografts were cultured for an additional 24 h prior to a second round of fluorescence imaging at 48 hpi for quantification.

### 2.6. Cell Culture and Labeling

A2058 cells and/or HUVECs were cultured in a 37°C cell culture incubator at 5% CO_2_. Tumor cells at a confluency of 60%–70% were labeled with CM-DiI for 20 min at 37°C, washed 3 times, 10 minutes with HBSS, and cultured overnight at 37°C. The next day, cells were trypsinized and collected for microinjection.

### 2.7. Total RNA Isolation and Quantitative Real-Time Polymerase Chain Reaction

Total RNA was extracted using the TRIzol reagent [12]; *vegfa*, *vegfr2*, *vegfr3*, *p53*, *casp3a,* and *18s* (supplemental data: Table S1) genes were used for quantitative real-time PCR. As an internal reference, 18S rRNA was used. The delta-delta Ct (2^−△△ CT^) method was used to calculate the relative change in gene expression.

### 2.8. Statistical Analysis

For statistical analysis, GraphPad Prism 5 software (GraphPad Software Inc., La Jolla, CA, USA) was used. Differences between groups were analyzed using one-way analysis of variance (ANOVA) followed by Tukey's post hoc test. Significance levels were set to ^*∗*^*p* < 0.05; ^*∗∗*^*p* < 0.01; and ^*∗∗∗*^*p* < 0.001.

## 3. Results

### 3.1. Inhibitory Effect of EEH or ECB on Melanoma-Induced Hyperplasia of Blood Vessels in Zebrafish Embryos

**Figure 1:**
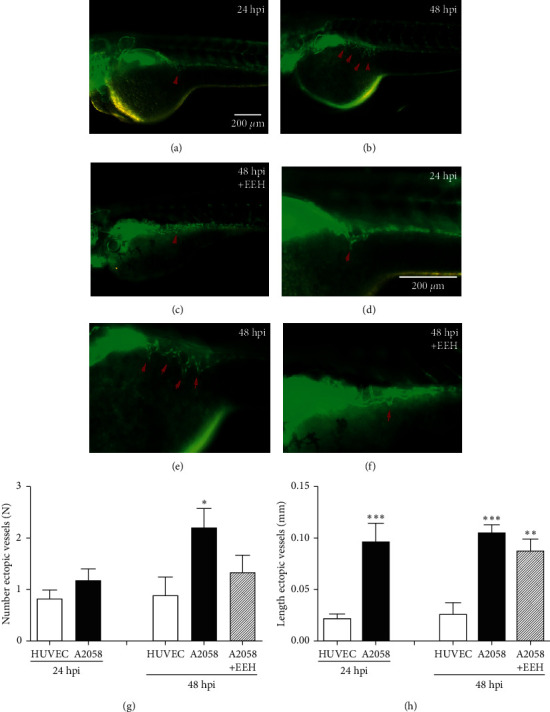
*Euphorbia ebracteolata* Hayata (EEH) inhibited SIV ectopic hyperplasia induced by melanoma cells in transgenic *Tg (flk1:GFP)* zebrafish embryos. Human melanoma cells were microinjected into zebrafish embryos at 48 hpf, and the resulting xenografts were imaged with a fluorescence microscope after 24 and 48 h (hpi), respectively. (a, d) SIV ectopic vessels in green fluorescence (red arrows) at 24 hpi zebrafish embryo (control). (b, e) SIV ectopic vessels (red arrows) at 48 hpi in zebrafish embryo (control). (c, f) SIV ectopic vessels after treatment with 50 *µ*g/mL EEH for 48 (h). (g) The length of ectopic blood vessels in A2058 or HUVEC xenografts at 24 and 48 hpi and the effects after treatment with *Euphorbia ebracteolata* Hayata (EEH) (^*∗*^*p* < 0.05). (h) The number of ectopic blood vessels in A2058 or HUVEC xenografts at 24 hpi and 48 hpi and the effects after treatment with EEH (^*∗*^*p* < 0.05). Scale bar = 200 *µ*m.

A2058 cells were microinjected beneath the surface of the yolk area at the 48 hpf stage in transgenic *Tg(flk1:GFP)* zebrafish embryos. At 24 hpi, 50 *μ*g/mL of EEH was added to the embryo medium of the zebrafish/melanoma xenograft, containing the injected cells labeled with red fluorescent dye (CM-DiI, Invitrogen). Tumor-induced angiogenesis was quantified by evaluating the number and length of ectopic vessels in the SIV of zebrafish/tumor xenografts at 24 and 48 h after injection (24 and 48 hpi) (Figure 1). The length of the SIV ectopic blood vessels in control HUVECs and A2058 cells was measured. Our data showed that the length of SIV ectopic blood vessels in the A2058 group was significantly higher than that in the control group at both 24 and 48 hpi (*p* < 0.001). In addition, treatment of xenografts with 50 *μ*g/mL EEH reduced the length of SIV ectopic blood vessels of A2058 xenografts. To verify the results on ectopic vessel length, we also quantified the number of ectopic vessels in each xenograft and found that EEH had a more clear effect on the inhibition of tumor angiogenesis in A2058 xenografts at 48 hpi (*p* < 0.05).

**Figure 2:**
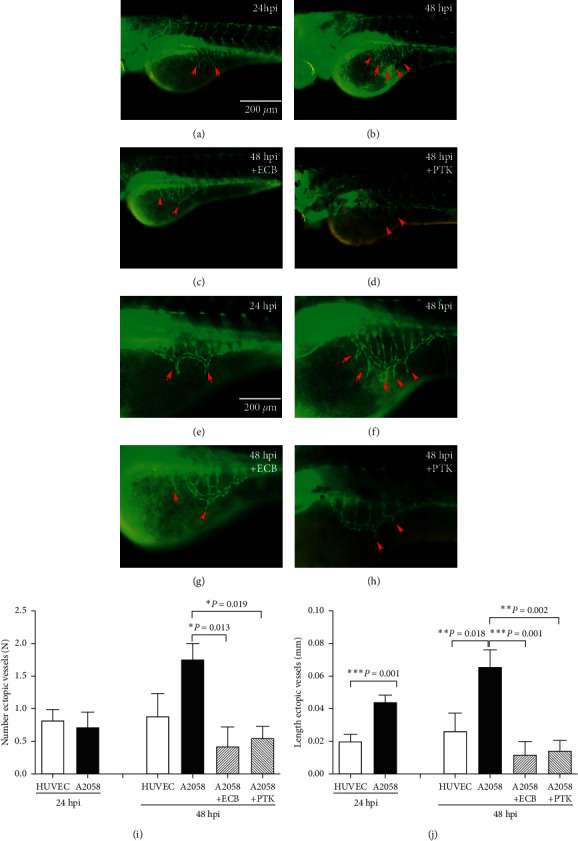
Euphorbiaceae compound B (ECB) inhibited SIV ectopic hyperplasia induced by melanoma cells in transgenic *Tg (flk1:GFP)* zebrafish embryos. Human melanoma cells were microinjected into zebrafish embryos at 48 hpf, and the resulting xenografts were imaged with a fluorescence microscope after 24 and 48 h (hpi), respectively. (a, e) SIV ectopic vessels in green fluorescence (red arrows) at 24 hpi zebrafish embryo (control). (b, f) SIV ectopic vessels (red arrows) at 48 hpi zebrafish embryo (control). (c, g) SIV ectopic vessels after treatment with 20 *µ*g/mL ECB for 48 h. (d, h) SIV ectopic vessels after treatment with 1 *µ*M PTK for 48 h. (i) The length of ectopic blood vessels in A2058 or HUVEC xenografts at 24 and 48 hpi and the effects after treatment with ECB and PTK (^*∗*^*p* < 0.05). (j) The number of ectopic blood vessels in A2058 or HUVEC xenografts at 24 hpi and 48 hpi and the effects after treatment with ECB or PTK (^*∗*^*p* < 0.05). Scale bar = 200 *µ*m.

Similar to EEH experiments at 24 hpi, 20 *μ*g/mL of ECB and 1 *μ*M of PTK were added to the embryo medium of the zebrafish/melanoma xenograft, and tumor-induced angiogenesis was quantified by evaluating the number and length of ectopic vessels in the SIV of zebrafish/tumor xenografts at 24 and 48 hpi (Figure 2). The length of SIV ectopic blood vessels in control HUVECs and A2058 cells was measured. Our data showed that the length of SIV ectopic blood vessels in the A2058 group was significantly higher than that in the control group at both 24 and 48 hpi (*p* < 0.01). In addition, treatment of xenografts with 20 *μ*g/mL ECB or 1 *μ*M PTK, which is antiangiogenic, significantly reduced the length of SIV ectopic blood vessels of A2058 xenografts (*p* < 0.001). Compared with control HUVECs, the length of ectopic vessels in both of ECB or PTK groups was not significantly different (*p* > 0.05) (Figure 2). Because the number of ectopic vessels was very small, it would be unable to compare the differences between treatments. To verify the results on ectopic vessel length, we also quantified the number of ectopic vessels in each xenograft and found that both ECB and PTK had a similar effect on the inhibition of tumor angiogenesis in A2058 xenografts at 48 hpi (*p* < 0.05). No significant differences were observed between A2058 cells and control HUVECs.

**Figure 3:**
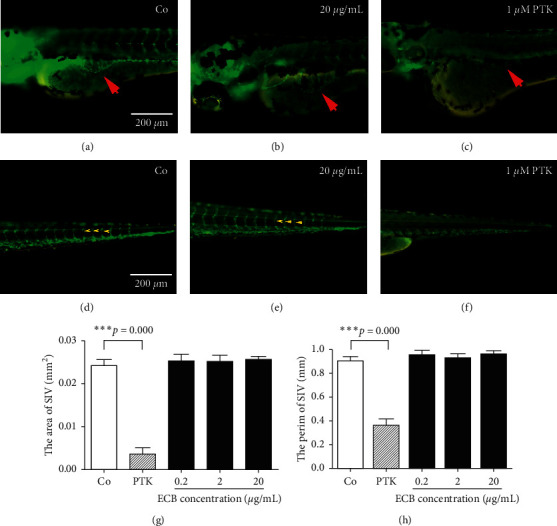
Effect of ECB on SIV and ISV angiogenesis in zebrafish embryos. Zebrafish embryos were imaged after treatment with ECB or PTK from 4 hpf to 72 hpf. (a, b) Control group. (b, e) ECB group. (c, f) PTK group. Red arrows point to SIV vessels above the yolk region (a–c). Yellow arrows show ISVs in the tail region (d–f). (g) The area covered by SIV vessels in zebrafish embryos (^*∗∗∗*^*p* < 0.001). (h) The perimeter (length) of SIV vessels in zebrafish embryos (^*∗∗∗*^*p* < 0.001). Scale bar = 200 *µ*m.

In embryos that were treated with different concentrations of ECB and PTK, no significant differences were observed at 72 hpf in perimeters or the area of the SIV between ECB groups and the control group. However, the positive control group that was treated with 1 *μ*M PTK showed reduction in both area and perimeter (length) of SIV blood vessels of the SIV compared with the control group/or the 20 *μ*g/mL ECB-treated group (*p* < 0.05). In addition, intersegment vessels (ISVs), as well as trunk artery and veins, were mostly missing in the 1 *μ*M PTK-treated group. Therefore, our findings suggested that ECB treatment did not affect normal angiogenesis in zebrafish embryos (Figure 3). This contrasted with the antiangiogenic molecule PTK that showed to be a potent inhibitor of all known VEGFRs (VEGFR-1, VEGFR-2, and VEGFR-3).

### 3.2. Effect of ECB on the Metastasis of Melanoma Cells in Zebrafish Xenografts

**Figure 4:**
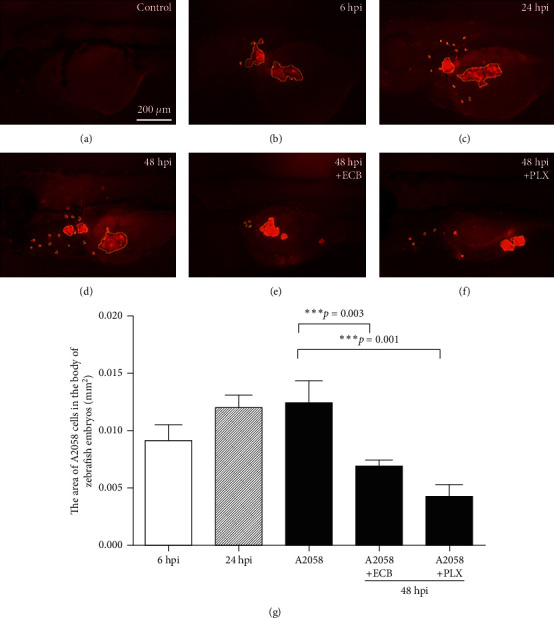
Effect of Euphorbiaceae compound B (ECB) on metastasis of melanoma cells in zebrafish xenografts. Zebrafish embryos were injected with A2058 cells at 48 hpf and observed at 6 hpi, 24 hpi, and 48 hpi, respectively. (a) Not injected. (b–d) A2058 cells were injected into zebrafish embryos at 48 hpf and observed at 6 hpi, 24 hpi, and 48 hpi, respectively. (e) A2058 cells were injected into zebrafish embryos and treated with 20 *µ*g/mL ECB for 24 h and observed at 48 hpi. (f) A2058 cells were injected into zebrafish embryos and treated with 1 *µ*M PLX4720 (PLX) for 24 h and observed at 48 hpi. (g) The area of CM-DiI labeling (melanoma cell) in the zebrafish embryos. Scale bar = 200 *µ*m. ^*∗∗∗*^ indicates a significant difference (*p* < 0.001).

The same number of melanoma cells (3 × 10^7^ cells/mL) was injected into the superficial region of the yolk sac after the zebrafish developed for 48 h. The metastatic effect of A2058 cells was observed in the zebrafish embryos at 6 hpi, 24 hpi, and 48 hpi (Figure 4). At 24 hpi, 20 *µ*g/mL ECB and the melanoma inhibitor PLX were added and zebrafish/tumor xenografts were analyzed after 24 h. The data showed that the area of CM-DiI labeling (melanoma cells) at 6 hpi, 24 hpi, and 48 hpi in zebrafish embryos was 0.009 mm^2^, 0.012 mm^2^, and 0.013 mm^2^, respectively. In zebrafish, the area of A2058 cells was reduced to 0.005 mm^2^ after treatment with 20 *µ*g/mL ECB, and the area of A2058 cells was reduced to 0.004 mm^2^ after treatment with 1 *µ*M PLX for 24 h (*p* < 0.001) (Figure 4(g)).

### 3.3. Effect of ECB on Blood Vessels and the Expression of Apoptosis-Associated Genes in a Zebrafish/Melanoma Xenograft Model

**Figure 5:**
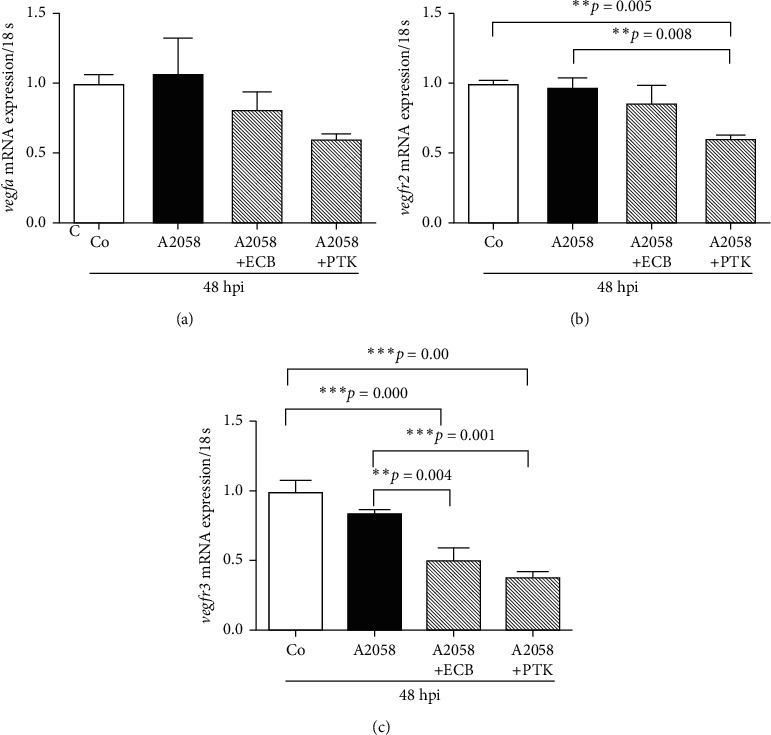
Effect of Euphorbiaceae compound B (ECB) on zebrafish *vegfa*, *vegfr2,* and *vegfr3* mRNA expression in xenografts using real-time PCR: (a) *vegfa* mRNA expression, (b) *vegfr2* mRNA expression, and (c) *vegfr3* mRNA expression. ^∗∗^ and ^∗∗∗^ indicate a significant difference (^*∗∗*^*p* < 0.01; ^*∗∗∗*^*p* < 0.001).

**Figure 6:**
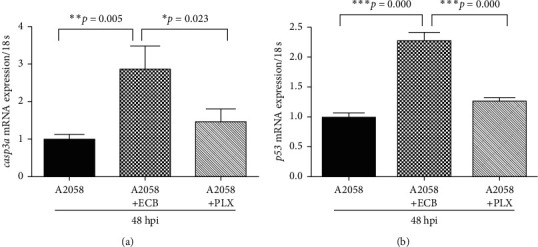
Euphorbiaceae compound B (ECB) affected the expression of zebrafish *casp3a* and *p53*mRNA in xenografts using real-time PCR. A2058 cells were labeled with CM-DiI, injected into zebrafish embryos at 48 hpf, and treated with 20 *µ*g/mL ECB and 1 *µ*M PLX or the medium only control at 72 hpf (24 hpi), respectively. *Casp3a* (a) and *p53* (b) mRNA expressions were quantified. ^*∗*^*p* < 0.05, ^*∗∗*^*p* < 0.01, and ^*∗∗∗*^*p* < 0.001.

A2058 cells were labeled with CM-DiI, injected into zebrafish embryos at 48 hpf, and treated with 0 (Control) or 20 *µ*g/mL ECB or 1 *µ*M PTK for 24 h from 72 hpf (24 hpi). Embryos were collected for the quantification of mRNA expression of *vegfa*, *vegfr2*, and *vegfr3* at 96 hpf (48 hpi) (Figure 5). The data showed that ECB or the vascular inhibitor PTK downregulated the mRNA expression of *vegfa*. Furthermore, PTK significantly downregulated the mRNA expression of *vegfr2*, which was 0.6-fold compared with that of the control group. Compared with the control group, ECB or PTK significantly downregulated the mRNA expression of *vegfr3* by 0.5-fold and 0.38-fold, respectively (*p* < 0.05). Furthermore, compared with the A2058 cells-injected group, ECB significantly increased the mRNA expression of *p53* by 2.3-fold and increased that of *casp3a* by 2.9-fold (*p* < 0.05). No significant changes were observed for PLX groups (*p* > 0.05) (Figure 6).

## 4. Discussion

To evaluate the inhibitory effect of EEH and ECB on melanoma, *Tg(flk1:GFP)* transgenic zebrafish embryos were used as an *in vivo* model. In brief, A2058 cells were labeled with red fluorescent dye and microinjected into 48 hpf zebrafish embryos. Therefore, the labeled cancer cells can be monitored for tumor growth and tumor-induced neovascularization in developmental zebrafish embryos. Injection of melanoma cells into zebrafish embryos not only resulted in the metastatic spread of melanoma cells but also induced angiogenesis (SIV) in zebrafish/tumor xenografts (Figure 1). Together, these results suggested that transplanted melanoma cells induced neovascularization in zebrafish/tumor xenografts. First, we found that EEH significantly inhibited the increase in length and number of ectopic blood vessels of the SIV. Next, we confirmed that ECB was the active ingredient of EEH and was more potent in inhibiting the length and number of ectopic blood vessels of the SIV and inhibited the migration of A2058 cells in zebrafish/melanoma xenografts. Furthermore, ECB reduced the mRNA expression of zebrafish *vegfa*, *vegfr2*, and *vegfr3* and induced the mRNA expression of zebrafish *p53* and *casp3a* in xenografts.

Remodeling of the vascular network was required to support the tissues needed for oxygen and nutrients during embryonic development [13]. The occurrence and metastasis of tumors increased the proliferation of blood vessels. Therefore, many anticancer drugs suppressed cancer by inhibiting angiogenesis [14]. Even for short-term treatment (1 h), the angiogenesis inhibitor SU5416 prevented new angiogenesis and angiogenic blood vessel formation. However, TNP470 required continuous exposure to block formation of the SIV and had no obvious effect on angiogenesis [15]. To screen chemical compounds, Manfred et al. made mitf::xmrk transgenic medaka. This was a stable transgenic melanoma model in which tumor development was observed in all gene carriers [16]. Here, we used a zebrafish/melanoma xenograft model to evaluate tumor-induced angiogenesis of the SIV in zebrafish embryos and showed that EEH or ECB inhibited the length of SIV ectopic blood vessels and reduced the number of blood vessels. Lenard et al. believed that the decrease in blood vessels was caused by cell self-fusion [13]. In addition, Koenig et al. suggested that Vegfa signaling can guide endothelial cells to migrate out of the existing vasculature and merge to form intestinal blood vessels. A similar mechanism may be used during angiogenesis in other organs [7]. Zhou et al. used the VEGFR1 antagonistic peptide F56 as an inhibitor to act on blood vessels and found that F56 did not affect VEGF-A-induced endothelial cell proliferation but did reduce endothelial cell migration and angiogenesis. In addition, F56 inhibited angiogenesis of the chorioallantoic membrane in chicken embryos and the SIV in zebrafish embryo. Monomeric peptide F56 has significant antitumor activity by inhibiting angiogenesis [17]. Ponatinib is a vascular inhibitor that is used for FDA-approved cancer treatment [18] and can also inhibit the formation of ISVs and the SIV in zebrafish embryos. The antiangiogenic effect of Ponatinib on HUVECs was evaluated using cell proliferation and migration, angiogenesis, and wound-healing assays. Ponatinib inhibited Vegf-induced phosphorylation of Vegfr2 and its downstream signaling, including the Akt/eNOS/NO pathway and the MAPK pathway (ERK and p38MAPK) [12]. In addition, 12-deoxyphorbol 13-palmitate in *Euphorbia fischeriana* Steud inhibited the Vegfr-2 signaling pathway, reduced microvessel density, inhibited VEGF, and blocked the PI3K/Akt/mTOR signaling pathway, thereby resulting in inhibition of MCF-7 breast cancer cell proliferation in mice [19, 20]. Similar to these findings, we found that ECB significantly inhibited the ectopic vascular length of the SIV in zebrafish and inhibited the mRNA expression of *vegf*, *vegfr2*, and *vegfr3* in zebrafish xenografts.

Jolkinolide B isolated from *Euphorbia fischeriana* Steud induced apoptosis in a B16F10 mouse melanoma model by altering glycolysis. In addition, jolkinolide B treatment increased mRNA expression of the apoptosis gene Bax, *Casp3,* and *Casp9,* reduced mRNA expression of antiapoptosis genes *Bcl2*, reduced the mitochondrial membrane potential of B16F10 cells, and increased the level of reactive oxygen species (ROS), thereby having antitumor effects [21]. Moreover, 12-deoxyphorbol 13-palmitate isolated from *Euphorbia fischeriana* Steud induced cell cycle arrest at the G2-M checkpoint of BGC823 cells and upregulated the expression of *p53*, *p21*, and *IκB-α* in tumor cells, leading to tumor cell apoptosis and the inhibition of tumor growth [22]. In this study, a zebrafish/melanoma xenograft model was established to demonstrate that ECB from *Euphorbia ebracteolata* treatment also inhibited proliferative blood vessels, which was associated with increased mRNA expression of *casp3a* and *p53* in zebrafish.

In this study, we investigated the inhibitory effect of EEH or ECB on hyperplasia of blood vessels caused by human melanoma cells in zebrafish embryos. EEH and ECB inhibited melanoma-induced vascular proliferation of the SIV in zebrafish. This inhibitory effect may be achieved by blocking zebrafish *vegfa* and mRNA expression of the Vegf receptors *vegfr2* and *vegfr3* that regulate blood vessels. The decrease in blood vessels may be affected by the apoptotic pathway. It is noteworthy that ECB treatment only inhibited abnormal blood vessel development induced by melanoma, which is different from vascular inhibitors that universally inhibit blood vessel growth, including normal angiogenesis. Taken together, these data show that ECB may have potential therapeutic value for anticancer treatment.

## Data Availability

The study data are available from the corresponding author upon request.

## Supplementary Materials

Supplementary Materials Graphic abstract: Scheme for EEH or ECB inhibition of SIV ectopic hyperplasia induced by melanoma cells in transgenic *Tg(flk1:GFP)* zebrafish/tumor xenograft embryos. Table S1. Primer for quantitative real-time PCR. Figure S1. HPLC chromatogram of EEH. A: EEH extracts, peak 8: ECB, 2,4-dihydroxy-6-methylbenzoidone, and B: Reference substance (EBC) (Bao, Y, 2014).
