# Supplementary material for: Inhibitory Effects of Euphorbia ebracteolata Hayata Extract ECB on Melanoma-Induced Hyperplasia of Blood Vessels in Zebrafish Embryos
Source: Evid Based Complement Alternat Med. 2021 Apr 26;2021:5543259. doi: 10.1155/2021/5543259 (PMC8096565; doi:10.1155/2021/5543259)
Supplement: Supplementary Materials — Graphic abstract: Scheme for EEH or ECB inhibition of SIV ectopic hyperplasia induced by melanoma cells in transgenic Tg(flk1:GFP) zebrafish/tumor xenograft embryos. Table S1. Primer for quantitative real-time PCR. Figure S1. HPLC chromatogram of EEH. A: EEH extracts, peak 8: ECB, 2,4-dihydroxy-6-methylbenzoidone, and B: Reference substance (EBC) (Bao, Y, 2014). [file 5543259.f1.docx]

The inhibitory effects of *Euphorbia ebracteolata* Hayata extract ECB on melanoma-induced hyperplasia of blood vessel in zebrafish embryos

Wenjing Dong^a^, Xinyue Han^a^, ChaoBao^a^, Saijilahu Tai^b^, Yuxia Bai^c^, Liang Xu^d^, Jingfeng Yang^a^, TinChung Leung^e^***, Wuliji Ao^c^**, Wu Dong^a^*

a Inner Mongolia Key Laboratory of Toxicant Monitoring and Toxicant and Toxicology, College of Animal Science and Technology, Inner Mongolia University for Nationalities, Tongliao, Inner Mongolia 028000, China

b Mongolian State University of Education，Ulaanbaatar 210648, Mongolia

c Inner Mongolia Research Institute of Traditional Mongolian Medicine Engineering technology/College of Mongolian Medicine and Pharmacy, Inner Mongolia University for Nationalities, Tongliao 028000, China

d Inner Mongolia Key Laboratory for the Natural Products Chemistry and Functional Molecular Synthesis, College of Chemistry and Chemical Engineering, Inner Mongolia University for Nationalities, Tongliao, Inner Mongolia 028000, China

e Julius L. Chambers Biomedical Biotechnology Research Institute, Dept. of Biological & Biomedical Sciences, North Carolina Central University, Kannapolis, NC 28081, USA.


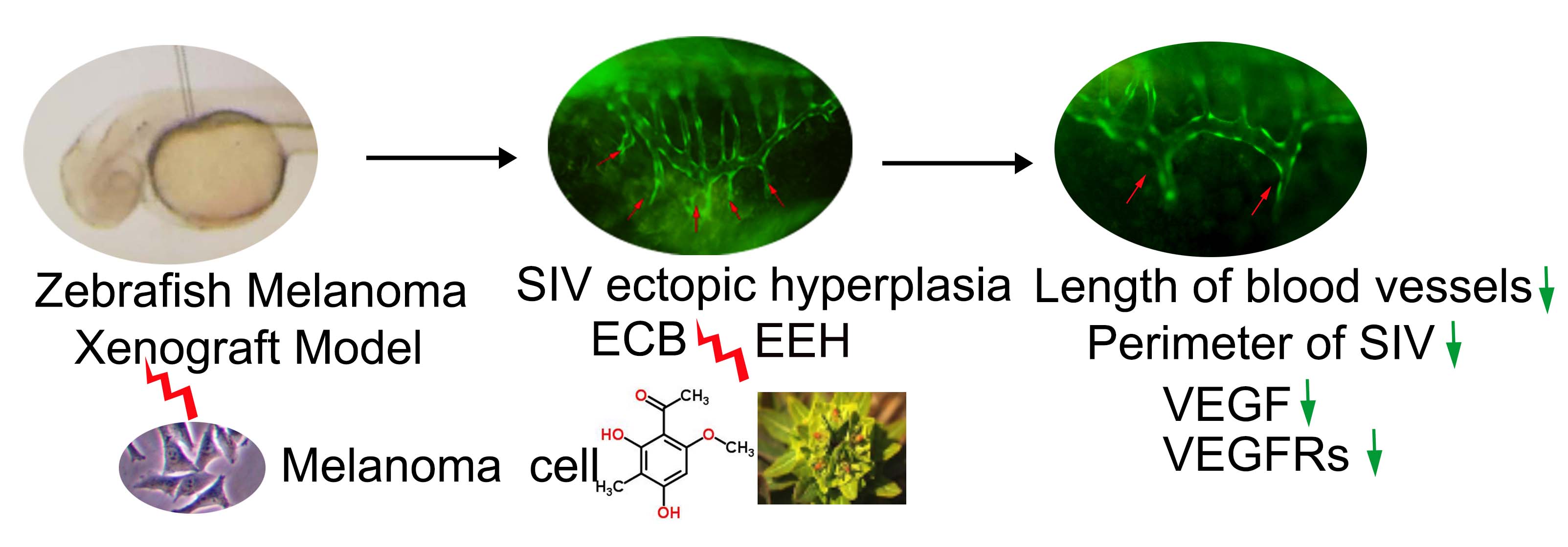


Graphic abstract:

Scheme for EEH or ECB inhibition of SIV ectopic hyperplasia induced by melanoma cells in transgenic *Tg(flk1:GFP)* zebrafish/tumor xenograft embryos.

Table S1. Primer for quantitative real-time PCR

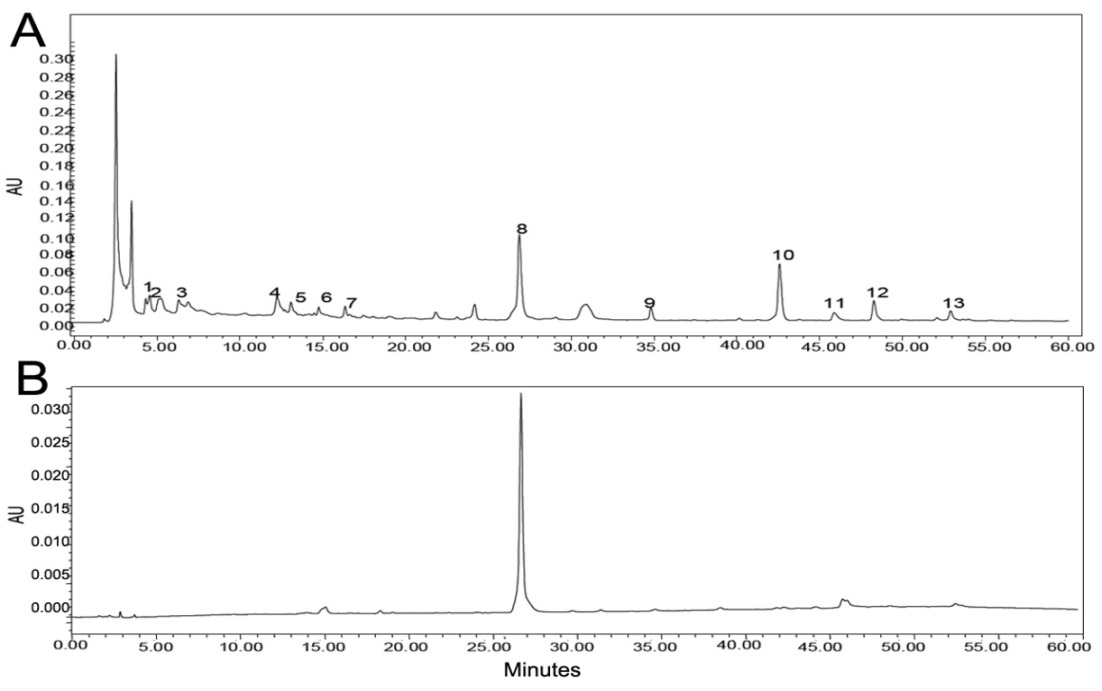


Figure S1. HPLC chromatogram of EEH. A: EEH extracts, peak 8: ECB, 2, 4-dihydroxy-6-methylbenzoidone, B: Reference substance (EBC). (Bao, Y, 2014)
